# Unveiling the relationship of the comorbidity between depression and type 2 diabetes mellitus: a macro analysis and micro interpretation

**DOI:** 10.3389/fmed.2026.1785271

**Published:** 2026-04-10

**Authors:** Donghai Wu, Yutian Chen, Genki Izumoji, Siying Qu, Yixiang Wang, Xiaofen He, Shuting Zhou, Yongliang Jiang, Haiju Sun, Xiaoyu Li

**Affiliations:** 1 The Third Affiliated Hospital of Zhejiang Chinese Medical University (Zhongshan Hospital of Zhejiang Province), Hangzhou, China; 2 Key Laboratory of Acupuncture and Neurology of Zhejiang Province, The Third Clinical Medical College, Zhejiang Chinese Medical University, Hangzhou, China; 3 The First Affiliated Hospital of Zhejiang Chinese Medical University (Zhejiang Provincial Hospital of Traditional Chinese Medicine), Hangzhou, China

**Keywords:** BDNF, bibliometric analysis, depression, HPA axis, T2DM

## Abstract

**Aims:**

This study aimed to address the lack of comprehensive reviews on the comorbidity between depression and type 2 diabetes mellitus (T2DM) by investigating their complex connections and identifying emerging research trends.

**Methods:**

We conducted an in-depth review combining macro bibliometric analysis and micro content interpretation of literature. Based on the bibliometric analysis of 3,986 papers in Web of Science Core Collection and 238 clinical trials in PubMed published between 2016 and 2025, the institutions, countries, authors and keywords were investigated, and the research maps were drawn by bibliometric software such as VOSviewer, Citespace and Bibliometrix. Meanwhile, micro interpretation entails in-depth content analysis of key articles to develop personal insights.

**Results:**

The analysis identified 3,986 relevant papers and 238 clinical trials, revealing a consistent overall growth in research volume. Key contributing entities and potential future research hotspots are mapped, such as the link between comorbidity and Parkinson’s disease and obesity. Also, our micro-interpretation identified the comorbidity mechanism through hypothalamic pituitary adrenal axis (HPA axis) and Brain derived neurotrophic factor (BDNF) and integrated treatment strategy of depression and T2DM.

**Conclusion:**

The results of the study outlined the evolving knowledge and research focus in this field. This study provides a comprehensive review of the comorbidity of depression and T2DM, emphasizing the mechanism and treatment of the comorbidity. The current research focus is on the HPA axis and BDNF, and the integrated treatment idea to solve depression and T2DM at the same time has the potential to change in chronic disease management and clinical practice.

## 1 Introduction

Depression and diabetes mellitus represent two most pressing global public health challenges in the 21st century. The comorbidity of psychological and physical physiological diseases constitutes a major public health issue. With the extension of life expectancy and various other socio-economic factors, the incidence of diseases continues to increase. There is a bidirectional association between psychological disorders and physical illnesses, and comorbidities increase the difficulty and complexity of diagnosis and treatment. Therefore, it is necessary to pay more attention to the simultaneous diagnosis and treatment of mental disorders and other related physical illnesses (1).

Depression, a debilitating mental disorder affecting over 280 million patients worldwide, is characterized by persistent sadness, cognitive impairment, and functional disability (2). Depression affects 2–21% of the general population and is ranked among top 10 causes of years lost to disability (3, 4). Also, depression can cause other mental diseases, such as anxiety and bipolar disorder, which can pose more serious harm to patients (5–7).

Although the exact etiology of depression remains in doubt, researchers have made great efforts to understand the complex mechanisms behind the multiple conditions of depression (8). From 1960 to the present, depression research is constantly being updated and scientists continue to study and treat depression from different perspectives (5). In the 1960s, depression was considered to be caused by the lack of norepinephrine mainly in the brain (9). In the 1990s, Selective Serotonin Reuptake Inhibitors (SSRIs) became a major breakthrough in the treatment of depression, which posited that reduced serotonin availability contributes to emotional dysregulation and cognitive impairments. These findings emphasize the role of serotonergic pathway in mediating therapeutic response, and stimulate the subsequent study of neural plasticity and epigenetic mechanisms of SSRI efficacy (10, 11). In the 21st century, the number of research on depression has reached a new peak and there is also an increasing amount of frontier research in this field. Currently, depression is considered the result of complex interaction between environmental factors, genetic factors, and neurobiological factors (12).

Meanwhile, diabetes, particularly type 2 diabetes mellitus (T2DM), is highly prevalent, with an estimated 537 million adults living with the condition in 2021, a figure projected to rise to 783 million by 2045 (13). Type 2 diabetes (T2DM) is a chronic metabolic disease, which is characterized by insulin resistance and the progressive decline of pancreatic beta cell function, leading to a sustained increase in blood sugar. It is the most common type of diabetes, accounting for more than 90% of global diabetes cases. Long term hyperglycemia in T2DM can lead to serious complications, such as diabetic retinopathy, diabetic nephropathy, stroke, hypertension and depression (14, 15). Depression is a common complication of T2DM, and there is a two-way relationship between the two diseases (16, 17).

Although these diseases are usually studied separately, current research has found a bidirectional relationship between T2DM and mood disorders, especially depression (18). A meta-analysis of 10 randomly controlled trials on T2DM shows a significantly higher trend of depression in patients with T2DM compared with those without (19). T2DM can lead to the development of depressive disorder, which is associated with higher T2DM complications and mortality rates (20). Epidemiological studies have also proved this: the risk of patients with depression developing T2DM increased by 60%, and the risk of depression in patients with diabetes is 2–3 times higher (21). This interplay exacerbates disease trajectories, leading to poorer glycemic control, increased complications, and elevated mortality rates.

Despite extensive studies, the mechanisms underlying this comorbidity remain elusive. Hypotheses range from shared biological pathways, such as chronic inflammation, hypothalamic pituitary adrenal axis (HPA axis) dysregulation (22), and gut microbiome alterations (23), to lifestyles (24) and psychosocial stressors (25). However, despite a substantial body of literature on the comorbidity of depression and T2DM, a systematic macroscopic overview, one that delineates the intellectual structure, evolution, and global collaboration patterns of this field, is conspicuously absent. Recent advances in bibliometric data visualization offer new opportunities to address this gap.

To address this gap, we conducted a bibliometric analysis and in-depth review. This study aligns with the United Nations Sustainable Development Goals (SDG 3.4) by addressing non-communicable disease multimorbidity (26). By transforming abstract epidemiological associations into intuitive visual narratives, this article visualizes past literature on comorbidity between depression and T2DM, making it clearer and more intuitive at a glance. Besides, an in-depth review was conducted, hoping that the relevant content can provide ideas for clinical research and future research directions.

Bibliometric analysis represents a valuable and purposeful approach for exploring scholarly literature within a specific domain (27). This methodology entails the examination of bibliographic information, including the annual volume of published articles, the frequency of citations they receive, and the collaborative networks among researchers (28). Through the application of statistical methods, bibliometric analysis enables the identification of highly influential researchers, the delineation of the evolution and transformations within academic disciplines, and the assessment of research productivity. Widely utilized in research assessment, institutional ranking, and the formulation of research-related policies, bibliometric analysis extends beyond evaluating individual researchers (29). It can also be employed to gauge the impact of research initiatives, funding bodies, and national research ecosystems. Among the primary tools used for bibliometric analysis are Citespace, VOSviewer and Bibliometrix, programmed by R studio, which facilitate data visualization and in-depth exploration of bibliometric data (30–32).

The data that is used for this bibliometric analysis comes from the Web of Science Core Collection and PubMed. Web of Science Core Collection is a multidisciplinary citation database that provides extensive coverage of high-level, peer-reviewed research articles or reviews spanning across different academic disciplines (33). PubMed is mainly used for analysis of current clinical research progress. By using these two databases, influential research works relevant to the analysis were identified.

In summary, this article conducts a bibliometric and visual analysis, coupled with an in-depth review, to explore the current understanding of the comorbidity between depression and T2DM. By integrating findings from various research fields, it reviews the internal mutual influences between the two diseases and presents treatment strategies, with a particular focus on the neurological perspective. The insights presented herein may offer valuable references for researchers in related disciplines and are expected to stimulate further academic inquiry.

## 2 Methods

### 2.1 Data acquisition

The data was obtained online from the Web of Science Core Collection (WoSCC) and PubMed.

The data search strategy in Web of Science was as follows: (TS = (depression) OR TS = (depressive) OR TS = (depressive disorder) OR (TS = (major depressive disorder) OR (TS = MDD))) AND (TS = (Type 2 Diabetes Mellitus) OR TS = (Type 2 Diabetes) OR TS = (T2DM) OR TS = (Diabetes Mellitus, Type 2)). The selection criteria were as follows: (1) language: English; (2) document type: article or review; and (3) timespan: 2016–2025. The date was collected in March 26th, 2025. Initially, there were 3,986 documents satisfied these criteria.

The data search strategy in PubMed was as follows: ((depression[Title/Abstract]) OR (depressive[Title/Abstract]) OR (depressive disorder[Title/Abstract]) OR ((major depressive disorder[Title/Abstract]) OR (MDD[Title/Abstract]))) AND ((Type 2 Diabetes Mellitus[Title/Abstract]) OR (Type 2 Diabetes[Title/Abstract]) OR (T2DM[Title/Abstract]) OR (Diabetes Mellitus, Type 2[Title/Abstract])). The selection criteria were as follows: Article type = (Clinical Trails), Language = (ENGLISH), timespan = 2016–2025. The date was collected in December 20th, 2025. Finally, 238 documents were found and analyzed.

**Figure 1:**
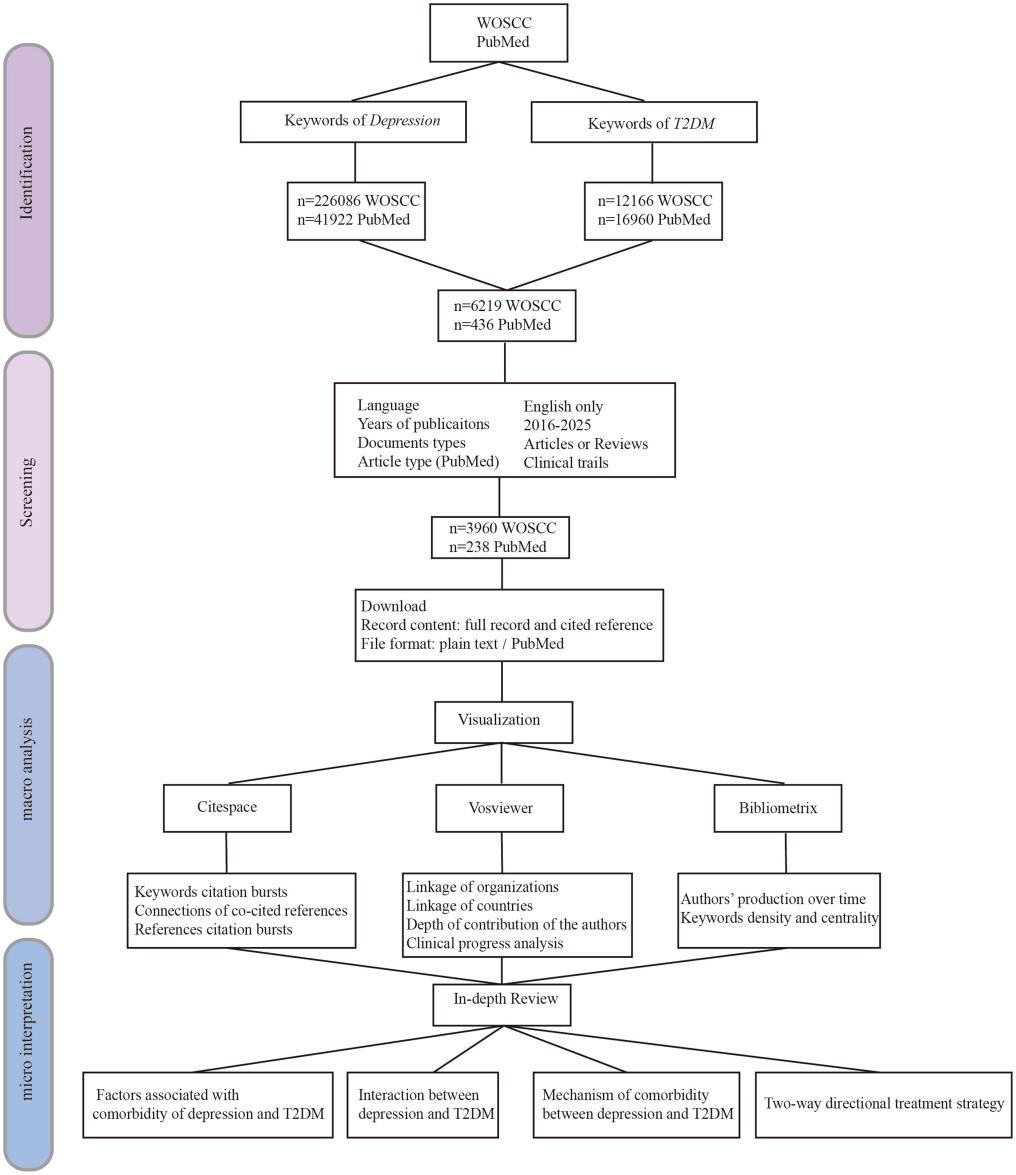
The flow chart of research methods in this review.

The specific methodology is presented in Figure 1, and it conforms to the reporting guideline PRISMA (Supplementary material).

### 2.2 Data analysis and visualization

After manually checking publications to avoid duplicate article searches and ensuring strict visualization analysis, the exported data is downloaded in plain text file format (WoSCC) or PubMed format, that includes complete records and references. Publications were extracted using Zotero 6.0.36. The data were then mainly imported into VOSviewer 1.6.19, The “Bibliometrix” package in the R environment 4.4.1, CiteSpace 6.2. R6, and GraphPad 9.5.1 for analysis. After comparing the data exported from each software with the data presented in the WoSCC, it can be confirmed that the data extracted by bibliometric software are accurate and the visualizations are reliable and valuable. Moreover, the data in PubMed is imported into VOSviewer to detect the burst of keywords.

VOSviewer is an excellent software specifically designed for the construction and visualization of bibliometric maps. This process provides valuable insights into the thematic and relational structures within the body of literature, facilitating a more comprehensive understanding of the research domain. In this article, it is used for showing the connection and density of countries, organizations, authors and keywords.

CiteSpace is a very effective tool in the field of scientific literature analysis and visualization. It excels in identifying emerging trends and pinpointing influential publications within a specific research domain. In this article, it is mainly used to detect the burst of keywords and references.

Bibliometrix is programmed by R Studio to perform comprehensive scientific mapping analysis. In this article, it is mainly used to display the changes of the authors’ productions over time and the centrality and density of keywords.

GraphPad Prism 9.5.1 was used to fit the trend line for the annual number of publications, visualizing the temporal evolution of research activity in this field.

## 3 Result

### 3.1 Publication years

**Figure 2:**
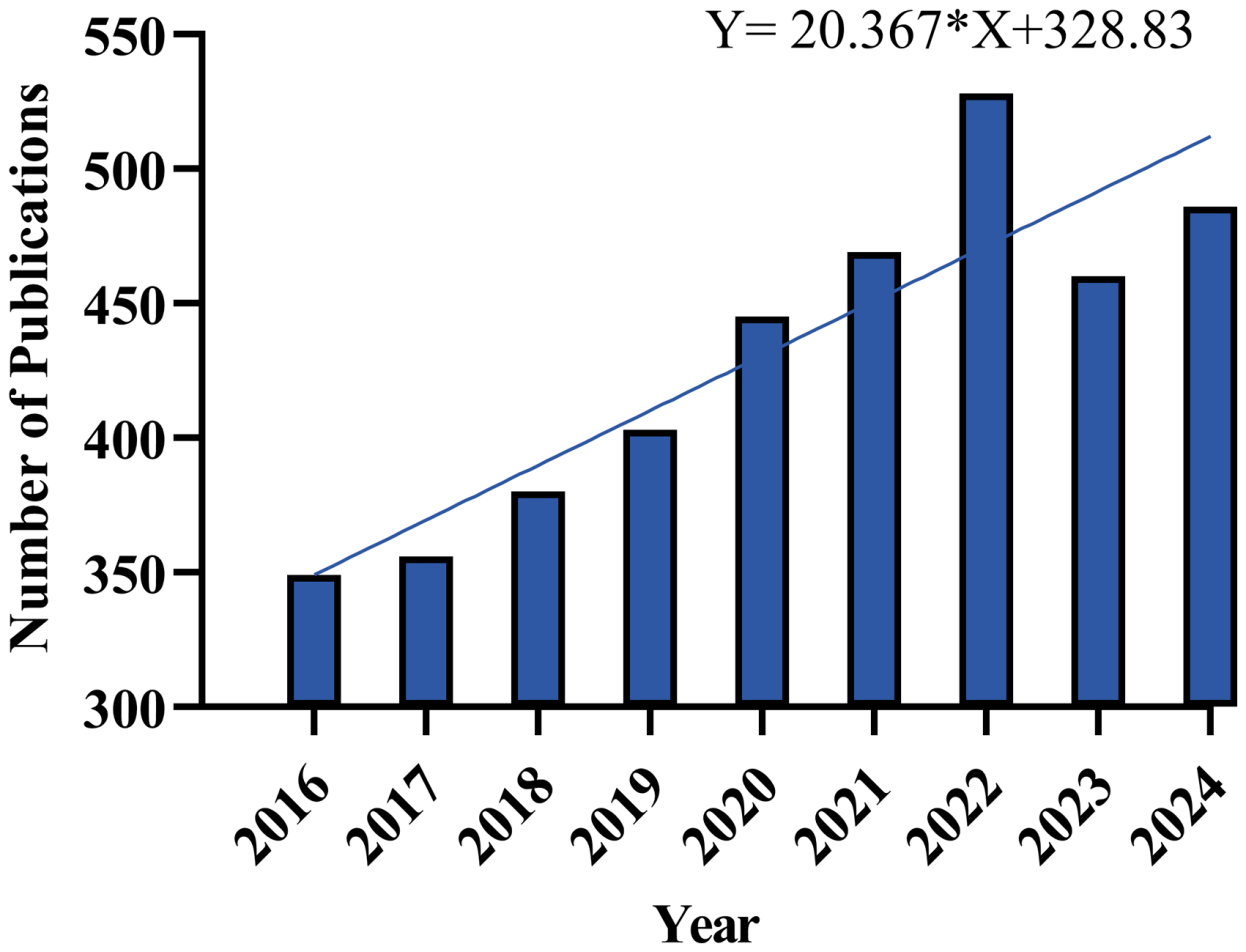
The number and trend of global publications.

Analyzing the time distribution of academic publications is helpful to reveal how a discipline evolves and to estimate whether it will be popular in the future. Between 2016–2025, annual publications centered on depression and T2DM totaled 3,986 articles, with trends in their annual output visualized in Figure 2, in which data for 2025 (*n =* 110) are not included.

In the last decade, the lowest number of publications was in 2016 (*n* = 349). With the output exceeding 400 publications each year since 2019, the annual publication count saw the steepest increase in 2022. While a slight decline occurred in 2023, the overall trend over the decade remains upward, indicating the field will likely maintain its scholarly interest and attract continued attention throughout the 2020s and beyond.

### 3.2 Organizations analysis

**Figure 3:**
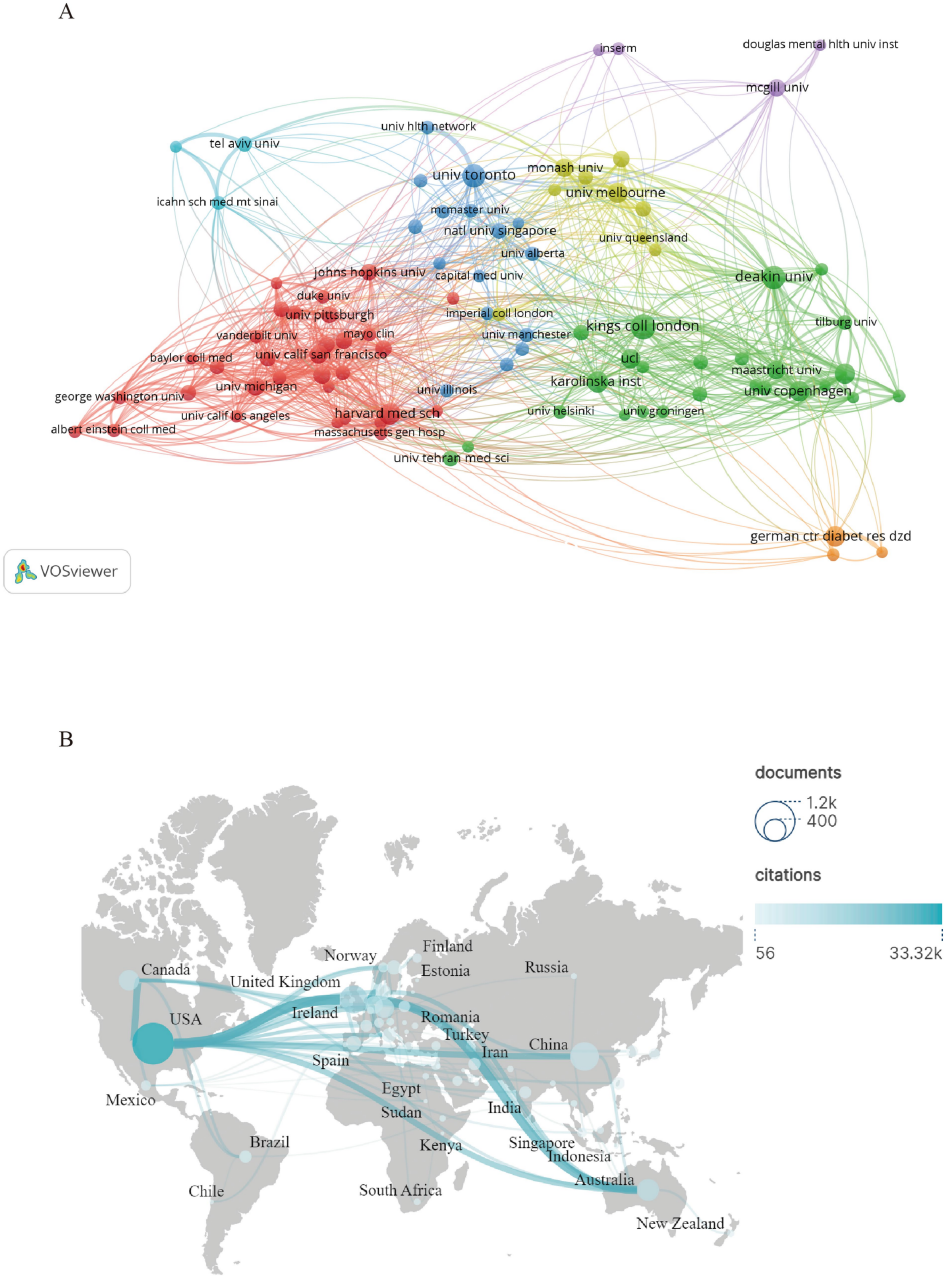
**(A)** The organizations linkage map generated by Vosviewer. **(B)** Map of countries’ collaboration hotspots.

Figure 3A presents a visualization generated through Vosviewer’s framework, which depicts the relationships between academic organizations through production of academic articles by institutions and inter institutional linkages factors. The quantitative analysis of academic contributions (Table 1) shows the top 20 most productive institutions and the citations of them, with King’s College London exhibiting the highest documents published, followed by Deakin University and the University of Toronto. These institutions have played a key role in interdisciplinary research on depression and T2DM. Their high ranking shows that these organizations have had a significant impact on the research direction and knowledge dissemination mode in this field.

**Table 1:** The quantitative analysis of academic contributions of top 20 organizations ranked by the number of documents.

Rank	Organization	Documents	Citations
1	King’s College London	88	2,887
2	Deakin University	75	2,419
3	University of Toronto	74	2,266
4	Karolinska Institute	67	2,724
5	Harvard Medical School	63	1759
6	University of Copenhagen	60	987
7	The University of Melbourne	58	1,659
8	University of Southern Denmark	58	1,135
9	German Center for Diabetes Research	57	1,072
10	University College London	53	1,401
11	University of Michigan	49	1,415
12	Maastricht University	48	1,588
13	University of California, San Francisco	48	1,408
14	Monash University	46	1,455
15	Stanford University	45	1,632
16	McGill University	44	617
17	University of California, San Diego	41	1,028
18	University of Pittsburgh	41	323
19	The University of Sydney	41	821
20	Johns Hopkins University	40	1,275

### 3.3 Countries or regions analysis

A total of 129 countries is represented in these documents, with 79 countries having published more than 5 documents. This distribution indicates a global research effort within the field. Figure 3B, generated using Scimago Graphica after data exporting from VOSviewer, visualizes the geographical relationships and connections, or can be explained as collaborative linkages, among countries. And Table 2 shows the top 10 countries or regions in terms of research productivity The USA (1,196 documents and 33,323 citations) leads with the largest number, also as the country which has the most highly-cited article published, followed at a considerable distance by China (572 documents and 8,310 citations), highlighting a strong growing research influence. Other notable contributors include the UK, Australia, Canada, Germany, Netherlands, Denmark, Spain, Sweden, all of which exhibit substantial research output and impact, as evidenced by the number of documents published and the associated citation counts.

**Table 2:** The top 10 countries or regions ranked by the numbers of citations.

Rank	Countries/Regions	Documents	Citations
1	USA	1,196	33,323
2	China	572	8,310
3	England	449	15,540
4	Australia	329	10,774
5	Canada	283	8,348
6	Germany	256	7,827
7	Netherlands	213	7,189
8	Denmark	161	5,505
9	Spain	154	4,273
10	Sweden	141	4,723

### 3.4 Authors analysis

**Figure 4:**
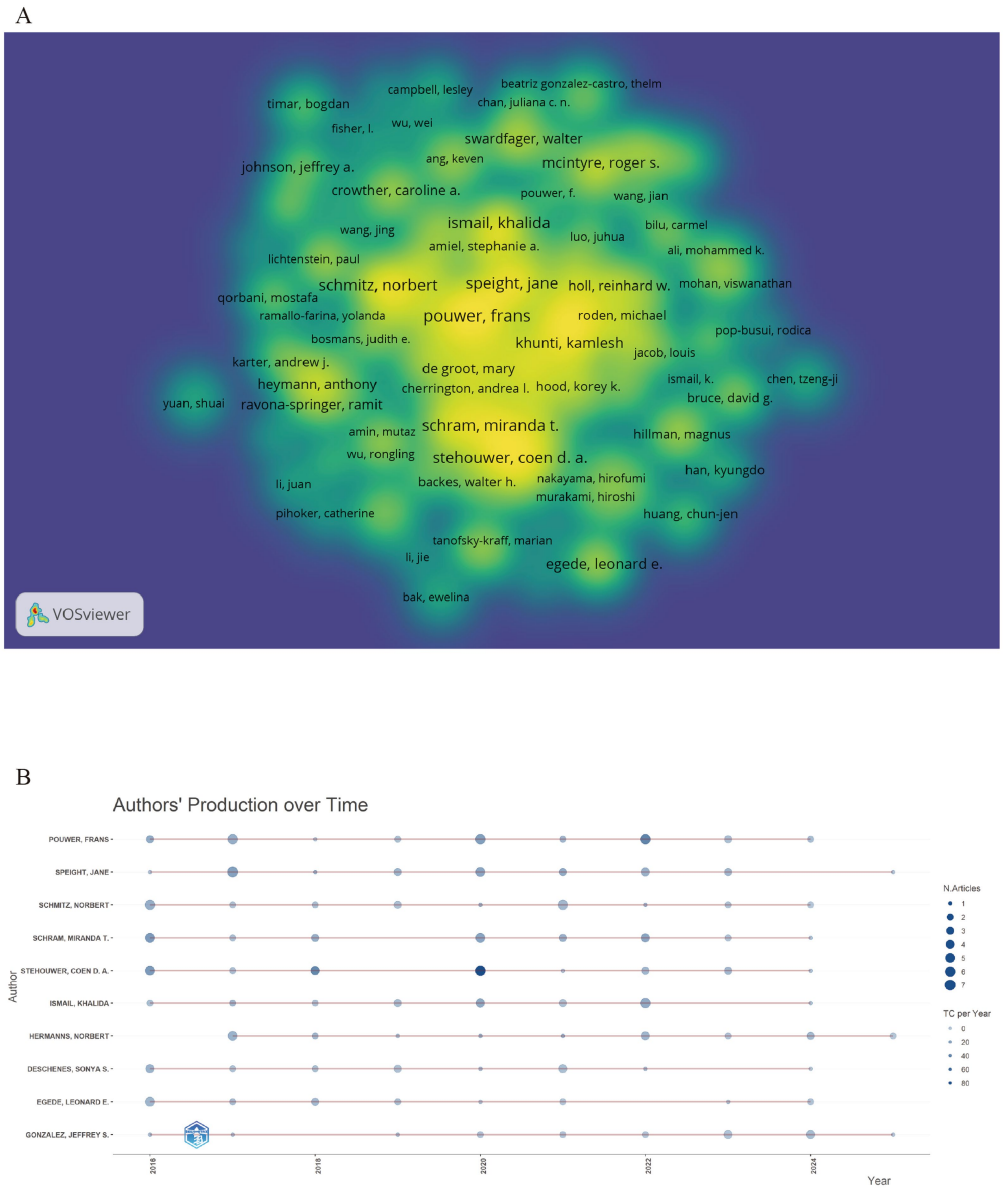
**(A)** Density view of author co-occurrence map generated by Vosviewer. **(B)** The map of top 10 authors’ production over time.

In total, 21,362 authors are associated with these documents, and among them, 309 of them have contributed 5 or more relevant articles. Frans Pouwer is the most cited authors with 90 citations. Figure 4A uses density bibliometric analysis to quantify individual academic contributions in the research field, while Table 3 quantitatively identifies 10 major authors through the number of documents published. Figure 4B depicts the top 10 authors’ production over time. Notably, most of them published consistently through the whole time period, not only underscoring their continuous research efforts, but also reflecting the in-depth knowledge of them in the field and the ability to adapt to evolving research trends.

**Table 3:** The top 10 authors ranked by the numbers of documents.

Rank	Author	Documents	Citations
1	Pouwer, Frans	30	757
2	Speight, Jane	28	673
3	Schmitz, Norbert	25	396
4	Schram, Miranda T.	25	729
5	Stehouwer, Coen D. A.	25	1,226
6	Ismail, Khalida	23	421
7	Hermanns, Norbert	21	415
8	Deschenes, Sonya S.	18	346
9	Egede, Leonard E.	18	279
10	Gonzalez, Jeffrey S.	18	147

### 3.5 Number of citations analysis

Citation analysis is an established quantitative indicator for evaluating academic impact within a professional research field. The universality of academic references to specific works is typically associated with three main attributes: innovative methods validated through peer recognition, empirical validity demonstrated through replicability, and theoretical significance reflected in the ability to shape paradigms. Table 4 summarizes the top 10 most frequently cited documents of the 3,986 documents searched based on citation analysis. The documents with the highest citation frequency and an impact factor of 98.4 is “Global, regional, and national incidence, prevalence, and years lived with disability for 310 diseases and injuries, 1990–2015: a systematic analysis for the Global Burden of Disease Study 2015” (34). This paper summarizes the analysis of the incidence rate, prevalence and years of disability survival (YLDs) of 310 diseases and injuries from 1990 to 2015 Global Burden of Disease (GBD 2015).

**Table 4:** The top 10 articles ranked by the number of citations.

Rank	First author	Article title	Citations	Journal	IF(2024)
1	Theo Vos	Global, regional, and national incidence, prevalence, and years lived with disability for 310 diseases and injuries, 1990–2015: a systematic analysis for the Global Burden of Disease Study 2015 (34)	2,905	Lancet	88.5
2	Ellulu, Mohammed S.	Obesity and inflammation: the linking mechanism and the complications (39)	1,216	Archives of Medical Science	3.3
3	Firth, Joseph	The Lancet Psychiatry Commission: a blueprint for protecting physical health in people with mental illness (40)	964	Lancet Psychiatry	24.8
4	Jodas Salvagioni, Denise Albieri	Physical, psychological and occupational consequences of job burnout: A systematic review of prospective studies (41)	760	PLoS One	2.6
5	Kunnumakkara, Ajaikumar B.	Curcumin, the golden nutraceutical: multitargeting for multiple chronic diseases (92)	717	British Journal of Pharmacology	7.7
6	Chekroud, Sammi R.	Association between physical exercise and mental health in 1.2 million individuals in the USA between 2011 and 2015: a cross-sectional study (93)	672	Lancet Psychiatry	24.8
7	Bellis, Mark A.	Life course health consequences and associated annual costs of adverse childhood experiences across Europe and North America: a systematic review and meta-analysis (94)	598	Lancet Public Health	25.2
8	Virani, Salim S.	2023 AHA/ACC/ACCP/ASPC/NLA/PCNA Guideline for the Management of Patients With Chronic Coronary Disease: A Report of the American Heart Association/American College of Cardiology Joint Committee on Clinical Practice Guidelines (95)	538	Circulation	38.6
9	Smith, Susan M.	Interventions for improving outcomes in patients with multimorbidity in primary care and community settings (96)	491	Cochrane Database of Systematic Reviews	9.4
10	Vancampfort, Davy; Correll, Christoph U.	Diabetes mellitus in people with schizophrenia, bipolar disorder and major depressive disorder: a systematic review and large scale meta-analysis (97)	485	World Psychiatry	65.8

### 3.6 Keywords analysis

**Figure 5:**
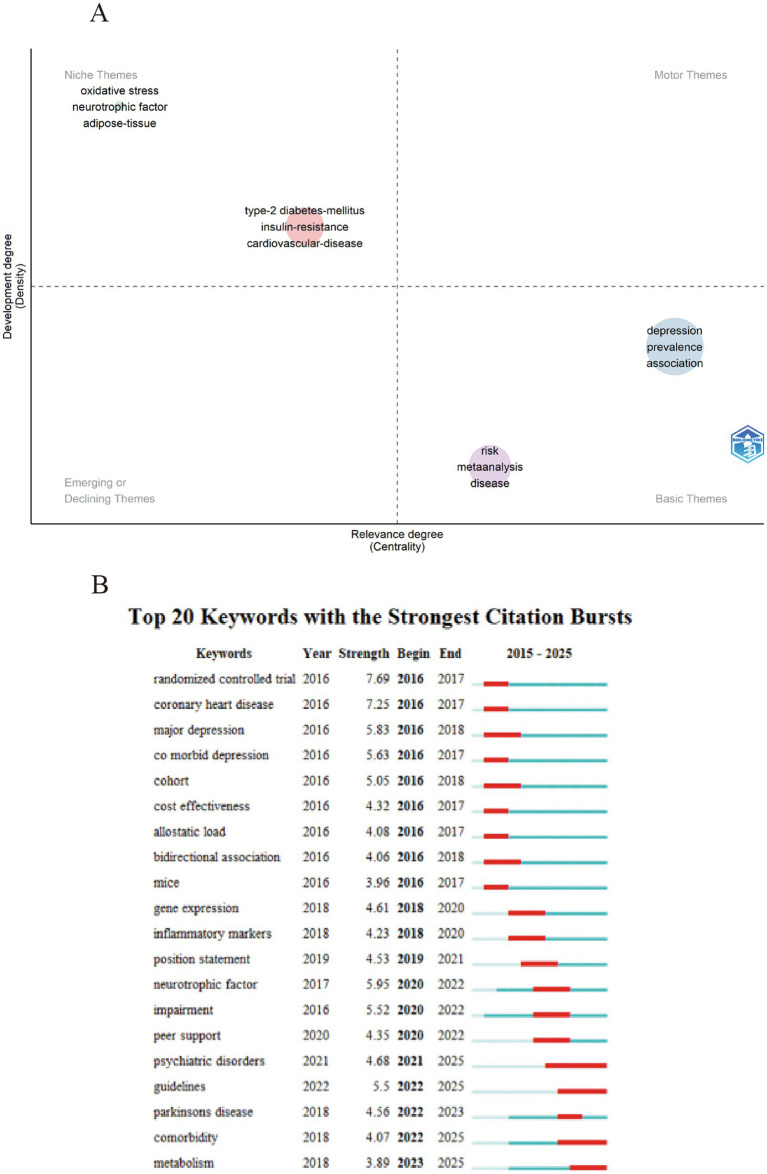
**(A)** The thematic map of keywords density and centrality. **(B)** Top 20 keywords with strongest citation bursts.

A total of 10,762 key terms were extracted from 3,986 documents. Among them, 134 key terms appear more than 50 times. The map in Figure 5A illustrates the keywords connections between the density and centrality. Table 5 lists the 20 most popular words, excluding manual related terms used in data retrieval, such as depression, type 2 diabetes, etc. The development of key terms is shown in Figure 5B. The surge in the citation of key terms can reflect the research focus of a discipline over a certain period of time and identify emerging themes. Citespace was used to identify keywords bursts in the literature on depression and T2DM from 2015 to 2025.

**Table 5:** The top 20 keywords ranked by the number of occurrences in WoSCC.

Rank	Keywords	Occurrences
1	prevalence	840
2	association	663
3	risk	646
4	adults	621
5	glycemic control	607
6	health	474
7	anxiety	472
8	symptoms	387
9	obesity	382
10	quality-of-life	364
11	people	355
16	meta-analysis	337
17	care	325
19	depressive symptoms	320
20	management	320

Keyword burst analysis reveals that the research landscape surrounding the comorbidity of depression and diabetes has undergone notable shifts while certain topics have remained consistently prominent. For instance the bidirectional relationship between these two conditions has been a sustained focus since the early stages (2016) of research in this field. Meanwhile the comorbidity of these diseases with other conditions such as Parkinson’s disease emerged as a research hotspot in 2023 and has since continued to gain attention.

### 3.7 Co-cited references analysis

**Figure 6:**
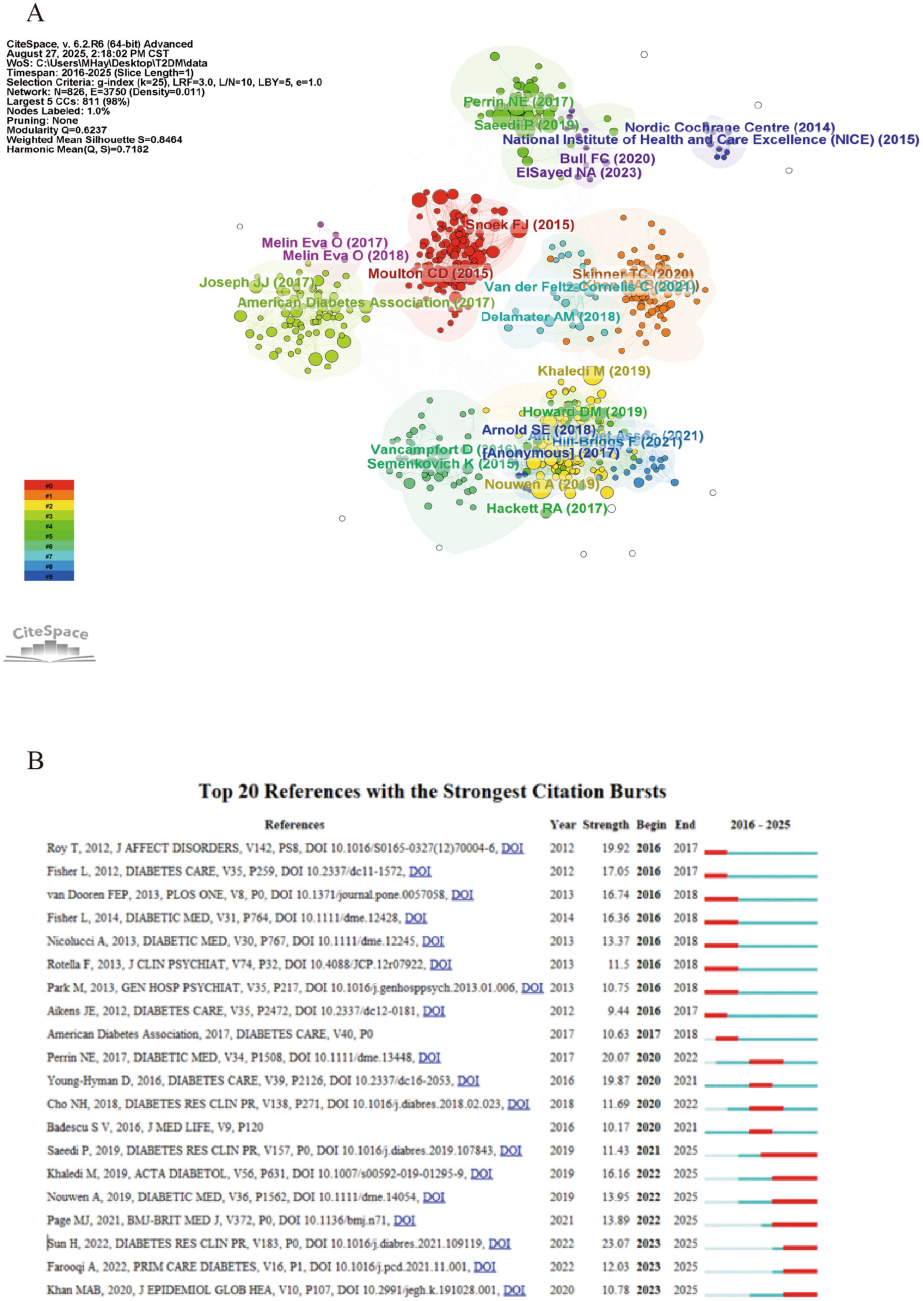
**(A)** The connections of co-cited references. **(B)** Top 20 references with strongest citation bursts.

Co-cited references refer to relevant works that repeatedly appear in the retrieved literature. Their repeated appearance indicates that these references elaborate on key theories or propose important concepts, providing substantial support for the field. Figure 6A shows the connections of the references. According to the evolution of research fields, Figure 6B shows the top 20 references in the development of depression and T2DM research from 2016 to 2025. Highly cited references often indicate a key turning point in the academic trajectory of the research field.

### 3.8 Clinical progress analysis

**Figure 7:**
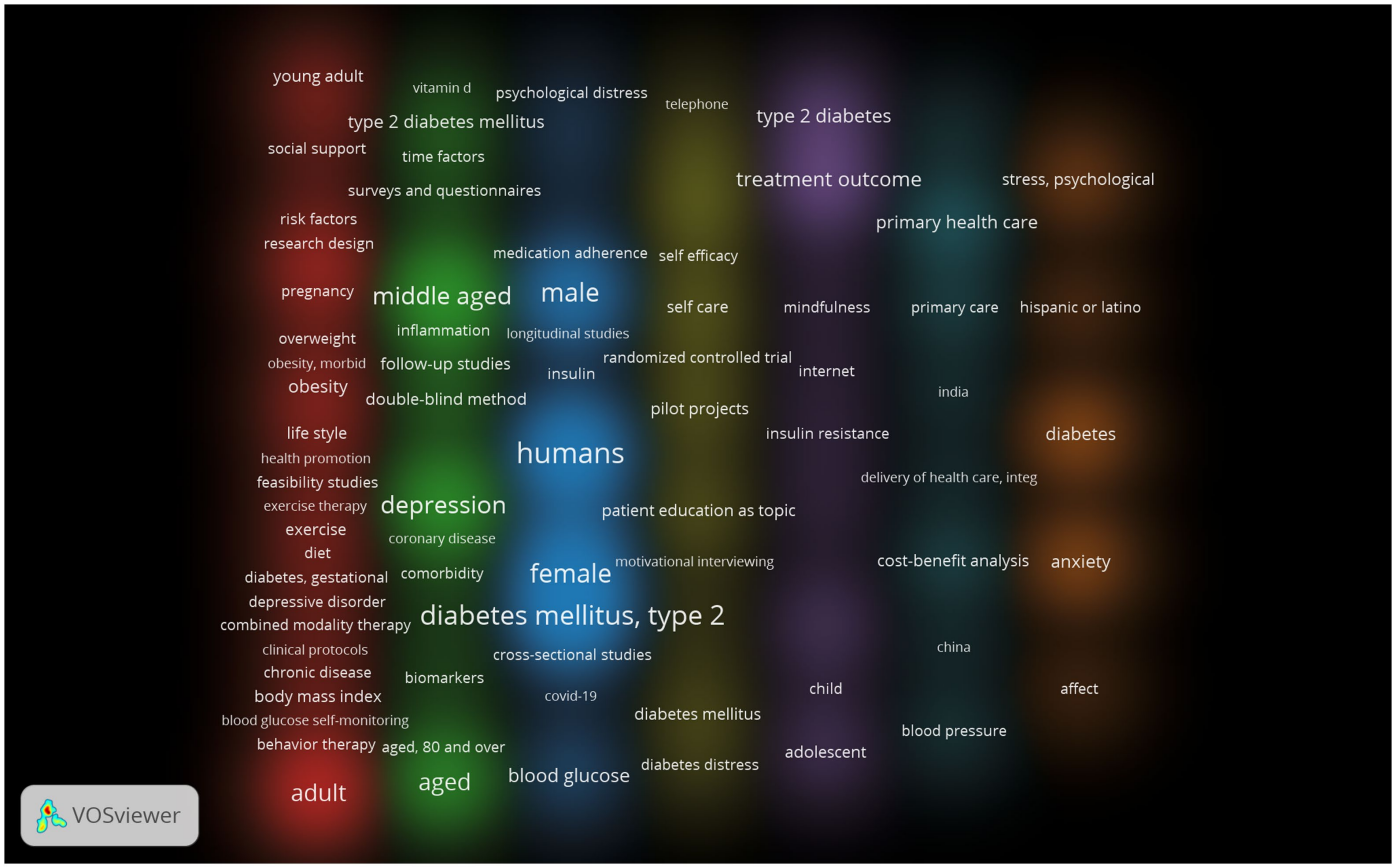
The keywords of clinical progress generated by Vosviewer.

PubMed search retrieved 238 clinical trial reports that met the inclusion criteria. PubMed independently verified the trend proposed by WoSCC, indicating that the research in this field is continuously deepening (Figure 7). Table 6 lists the top 10 keywords with the highest frequency after manually removing the words related to the search words. The clinical significance of comorbidity of depression and type 2 diabetes mellitus is worth further exploration.

**Table 6:** The top 20 keywords ranked by frequency of occurrence in PubMed.

Rank	Keywords	Occurrences
1	humans	236
2	middle aged	149
3	adult	111
4	aged	99
5	glycated hemoglobin	70
6	treatment outcome	61
7	quality of life	54
8	blood glucose	38
9	anxiety	34
10	self-management	26
11	obesity	25
12	primary health care	25
13	cognitive behavioral therapy	24
14	double-blind method	23
15	adolescent	22
16	self care	21
17	stress, psychological	21
18	follow-up studies	20
19	weight loss	20
20	mental health	19

It is worth noting that obesity has become one of the most valuable keywords, reflecting its dual role as a common risk factor and the potential mechanism between depression and type 2 diabetes. This emphasizes the importance of comprehensive weight management strategy in future research and clinical practice for this comorbidity.

## 4 Discussion

### 4.1 General information

Abundant evidence highlights a bidirectional relationship between T2DM and depression, with both conditions increasing the risk and severity of the other. The prevalence of depression in T2DM patients was significantly higher than that in the general population. Diabetes significantly increases the risk of depression. A study was found that 40% of T2DM patient have depression (35). Moreover, the incidence of depression in type 2 diabetic patients was higher than that in non-diabetic patients (36). The risk of T2DM in patients with depression increased by 37–60% (37). Consequently, the comorbidity of T2DM and depression amplifies adverse clinical outcomes.

### 4.2 Global trend

Analysis of research institutions and countries or regions reveals that studying the comorbidity of depression and T2DM necessitates a collaborative effort, as it cannot be accomplished by a single institution or region. The United States and China lead in the number of publications on this topic, suggesting that these two countries have likely invested significant scientific research resources into exploring the relationship between depression and T2DM. The high volume of published work reflects the substantial research capabilities of the United States and China within the field of depression neuroscience. Their research initiatives cover a broad spectrum, encompassing fundamental neurobiology, endocrinology, and clinical treatment. This comprehensive approach has provided a robust scientific foundation for deepening our understanding of depression and devising more effective treatment strategies. Given the global significance of depression as a mental health problem and the large number of patients worldwide affected by T2DM, not only adults, but also a large number of children, it is not surprising that researchers in China and the United States have paid extensive attention to this field and conducted in-depth investigations (38).

Figure 3B depicts the geographical distribution pattern of major academic entities engaged in the investigation of comorbidity between depression and T2DM. An important observation can be seen from the institutional ranking: the top 5 outstanding research organizations come from different national backgrounds and span across 3 continents. This geographical dispersion reveals a contradictory trend in the development of the discipline, although there are persistent differences in resource allocation and research infrastructure among regions, the intellectual importance of this interdisciplinary field indicates cross-border diffusion. This field has not shown a centralized cognitive advantage, but rather a multi-center development characterized by transnational research networks. These dispersed but interconnected academic centers promote knowledge synthesis through comparative epidemiological research and cross-cultural intervention trials, thereby accelerating the maturity of evidence-based frameworks for managing this complex dual pathology. The inclusion of institutions from various countries in the top 10 rankings creates a foundation and opportunity for international research collaboration. It encourages the diversification of research perspectives, enabling countries to engage in cooperative research by leveraging their respective strengths, integrating resources, technologies, and talent, with the aim of achieving more significant breakthroughs in the study of depression and T2DM comorbidity.

The global research pattern of comorbidity between depression and T2DM reveals two key characteristics: cross-border cooperation and knowledge integration. Analysis shows that leading institutions in countries are jointly driving progress through shared methods and comparative clinical research. Although regional research capabilities vary, the development of this field now depends on international partnerships that combine different medical perspectives and epidemiological data. This collaboration has achieved three key processes: standardization of diagnostic criteria, implementation of joint clinical trials, and coordinated data reporting systems. Such cooperation is indispensable for addressing challenges posed by the complex nature of comorbidity and the underlying mechanisms linking depression and T2DM. This shift highlights the situation from isolated national projects to interconnected research frameworks that address complex chronic disease interactions.

### 4.3 Analysis of highly cited articles searched

In the result, the most highly cited article, “Global, regional, and national incidence, prevalence, and years lived with disability for 310 diseases and injuries, 1990–2015: a systematic analysis for the Global Burden of Disease Study 2015” is identified, which provides a systematic analysis of disease incidence and prevalence with annual updates (34). Meanwhile, other highly cited articles identified in this bibliometric analysis also warrant in-depth interpretation.

“*Obesity and inflammation: the linking mechanism and the complications*” mainly discusses the correlation mechanism between obesity and inflammation, and how these mechanisms lead to metabolic diseases and cardiovascular diseases. This article emphasizes the key mechanism of obesity leading to diabetes and cardiovascular disease through inflammation and metabolic abnormalities (39).

*“The Lancet Psychiatry Commission: a blueprint for protecting physical health in people with mental illness”* published in Lancet Psychiatry. This article summarizes the results of nearly 100 systematic reviews and meta-analysis, and emphasizes the close relationship between mental illness and cardiovascular metabolic diseases, such as obesity, diabetes and cardiovascular disease. Depression is a common mental disease, which is closely related to physical health problems. Research shows that the obesity rate of patients with depression is significantly higher than that of the general population. Obesity will not only affect mental health, but also reduce the quality of life of patients, and may even lead to the decline of treatment compliance. In addition, the prevalence of metabolic syndrome (including diabetes and cardiovascular disease) in patients with depression also increased significantly. Obesity and metabolic syndrome may further affect the course of depression through inflammatory mechanisms. Diabetes is one of the common physical health problems in patients with mental illness. Research shows that the risk of diabetes in patients with mental illness is 1.4 to 2.0 times that of the general population. For patients with mental illness, the management of diabetes needs special attention. Metformin is still the first-line drug, which can avoid the transition to type 2 diabetes, but other drugs, such as glucose like peptide 1 receptor, can also be used to control blood glucose and weight, and avoid the deterioration of the disease (40).

*“Physical, psychological and occupational consequences of job burnout: A systematic review of prospective studies”* aims to summarize the impact of job burnout on physical, psychological and professional aspects through a systematic review of prospective research. Both depression and diabetes are associated with burnout. Burnout is highly correlated with depression. In the statistics of this article, the correlation caused by the similarity of the two measurement methods is excluded, which proves that the two are different cases. Meanwhile, this paper proves that burnout will make people more frequently engage in unhealthy behaviors, such as poor or rich diet, lack of physical exercise and alcohol abuse. When combined with sleep disorders, it will eventually lead to obesity and diabetes (41).

The chronic inflammation is identified as the key mechanistic link. Depression is highly correlated with obesity, which in turn drives metabolic abnormalities and inflammation, ultimately leading to the development of diabetes and cardiovascular diseases. Burnout, a critical upstream ignition points for this process. It is closely related to depression, and can cause diabetes and obesity by causing frequent unhealthy behaviors. These psychological and behavioral factors converge to cause obesity, thereby activating the inflammatory mechanism described as mentioned previously. Finally, The Lancet Psychiatry Commission describes the tangible clinical outcome and the resulting vicious cycle. It provides epidemiological evidence confirming the high co-morbidity rates between depression and diabetes, demonstrating the real-world consequence of the pathways outlined in the paragraphs above.

In conclusion, these three articles form an interconnected model: Burnout, or taking it as acts as pressure, a common trigger, leading to depression and unhealthy behaviors, which promote obesity. Obesity then fuels chronic inflammation, which is the core mechanism driving the onset of diabetes. Ultimately, these conditions lock into a self-perpetuating cycle that worsens both mental and physical health outcomes.

### 4.4 Multiple factors associated with comorbidity of depression and T2DM

The comorbidity of depression and T2DM includes not only physiological factors, but also many related psychological and social factors (42). The psychological, behavior and social factors include negative life events (43), lack of daily physical activities (44), being a female, and maintaining a single status, including widowhood or divorce (45).

T2DM patients face long-term pressure in disease management, including blood glucose monitoring and dietary control, compounded by life events, and may also face discrimination due to disease labels such as “laziness” and “poor self-control.” These issues have increased their likelihood of developing depression (46). Meanwhile, T2DM patients with depression often experience decreased willpower, such as unwilling to take medication or ignoring dietary management, leading to uncontrolled blood sugar levels, further aggravating diabetes (47). Consequently, Depression and T2DM leading to bidirectional deterioration in psychological and social factors.

### 4.5 The interaction between depression and T2DM

#### 4.5.1 Effect of depression on T2DM

Depression induces chronic stress response by activating the hypothalamus pituitary adrenal axis (HPA axis), leading to the increase of glucocorticoids (such as cortisol) (48). Cortisol can inhibit insulin signaling pathway (such as PI3K/Akt pathway) and aggravate insulin resistance by binding with glucocorticoid receptor (GR) (49, 50). In addition, cortisol promotes hepatic gluconeogenesis and lipolysis, leading to the increase of free fatty acids and further impairing insulin sensitivity (51).

At the same time, depression can enhance the risk of T2DM through chronic inflammatory response (52). The levels of pro-inflammatory cytokines (such as IL-6 and TNF-*α*) in the peripheral blood of patients with depression are increased. These inflammatory cytokines affect T2DM through the following ways: TNF-α activates JNK and NF-κB pathways and interferes with the phosphorylation of insulin receptor substrate (IRS). Inflammation promotes the release of free fatty acids and adiponectin by adipocytes, and aggravates insulin resistance. Inflammatory factors directly inhibit the function of islet *β* cells and induce apoptosis (53).

#### 4.5.2 Effect of T2DM on depression

Insulin resistance is one of the characteristics of T2DM. Insulin resistance not only leads to high blood sugar, but also affects insulin signaling in the brain, causing neuronal energy metabolism disorders and impaired synaptic plasticity. This metabolic disorder is closely related to the neurobiological mechanisms of depression, such as a decrease in monoamine neurotransmitters (54). The decrease of insulin sensitivity in T2DM patients is positively related with the severity of depression, indicating that metabolic abnormalities directly affect emotional regulation (55). Meanwhile, long term hyperglycemia triggers injury of hippocampus and neurodegeneration by activating the polyhydroxy pathway and oxidative stress, impairing memory and emotion regulation functions (56, 57). Reduced hippocampal volume has been reported in both T2DM and major depressive disorder (MDD) patients, which may be the structural basis for their comorbidity (58, 59).

Additionally, the long duration of diabetes imposes a significant burden and difficulties in daily life of patients, which may increase susceptibility of patients to depression (60–62).

### 4.6 Neuroendocrine regulation and molecular mechanism of comorbidity between depression and T2DM

#### 4.6.1 The HPA axis mechanism of comorbidity between depression and T2DM

Chronic stress often accompanies depression and T2DM, continuously activating the HPA axis and leading to increased secretion of corticotropin releasing hormone (CRH) and cortisol (63). The hyperglycemia status of T2DM patients further exacerbates HPA axis activity, forming a vicious cycle (22). Long term high cortisol levels lead to impaired glucocorticoid receptor (GR) function, ineffective negative feedback inhibition, sustained HPA axis activity, and high cortisol levels (64). This resistance not only exists in the central nervous system, but also affects peripheral tissues such as immune cells and the liver, exacerbating insulin resistance and inflammatory reactions (65). These conditions further exacerbate the occurrence of depression and T2DM. Hyperglycemia in T2DM patients activates the sympathetic adrenal medulla axis (SAM axis) and immune system, promoting the release of pro-inflammatory factors such as IL-1 *β*, IL-6, TNF-*α*, etc (22). These cytokines can directly stimulate hypothalamic CRH neurons, further activating the HPA axis (66). Chronic inflammation exacerbates insulin resistance and hyperglycemia, which in turn promotes oxidative stress and mitochondrial dysfunction, forming a vicious cycle of the “metabolism inflammation HPA axis” that jointly drives the development of depression and T2DM (67).

In summary, the HPA axis mechanism is as follows. Chronic stress and hyperglycemia leading to excessive activation of the HPA axis, elevated cortisol levels, triggering GR resistance, and negative feedback failure; Immune dysfunction and increased levels of pro-inflammatory cytokines further stimulate the HPA axis; High cortisol exacerbates insulin resistance and T2DM, while causing nerve damage and neurotransmitter imbalance, promoting depression.

#### 4.6.2 BDNF in comorbidity between depression and T2DM

Brain derived neurotrophic factor (BDNF) plays multiple roles in the comorbidity of depression and T2DM, and its influence mechanism involves neural plasticity, metabolic regulation and genetic susceptibility. BDNF levels are significantly reduced in patients with depression and are correlated with the severity of symptoms (68, 69). Antidepressant treatments, such as medication and electroacupuncture, can improve symptoms by increasing BDNF levels (70). The serum BDNF levels of T2DM patients with depression also significantly decrease, accompanied by metabolic abnormalities such as high cortisol and shortened telomeres (71). This low BDNF state may exacerbate both neuronal damage and metabolic disorders, forming the basis for comorbidity (72). Overexpression of BDNF can restore the activity of the neural pathway from the ventral inferior subiculum (vSub) to the bed nucleus of the anterior stria terminalis (aBNST) of the brain in diabetes, reduce the susceptibility to stress, and thus alleviate depressive symptoms (72). BDNF inhibits diabetes related atherosclerosis by promoting M2 macrophage polarization, and affects insulin secretion and glucose metabolism. Low levels of BDNF may simultaneously disrupt neuroprotection and metabolic homeostasis, leading to worsening of comorbidities (71, 72).

In summary, the impact of BDNF in comorbidities of depression and T2DM may involve the following points: (1) a common decrease in BDNF levels, leading to decreased neural plasticity and abnormal metabolic regulation; (2) BDNF gene polymorphism (such as Val66Met) increases the comorbidity risk of two diseases; (3) Metabolic abnormalities such as high blood sugar further inhibit BDNF and exacerbate depressive symptoms; (4) BDNF affects the development of two diseases by regulating inflammation, stress response, and insulin signaling pathways.

**Figure 8:**
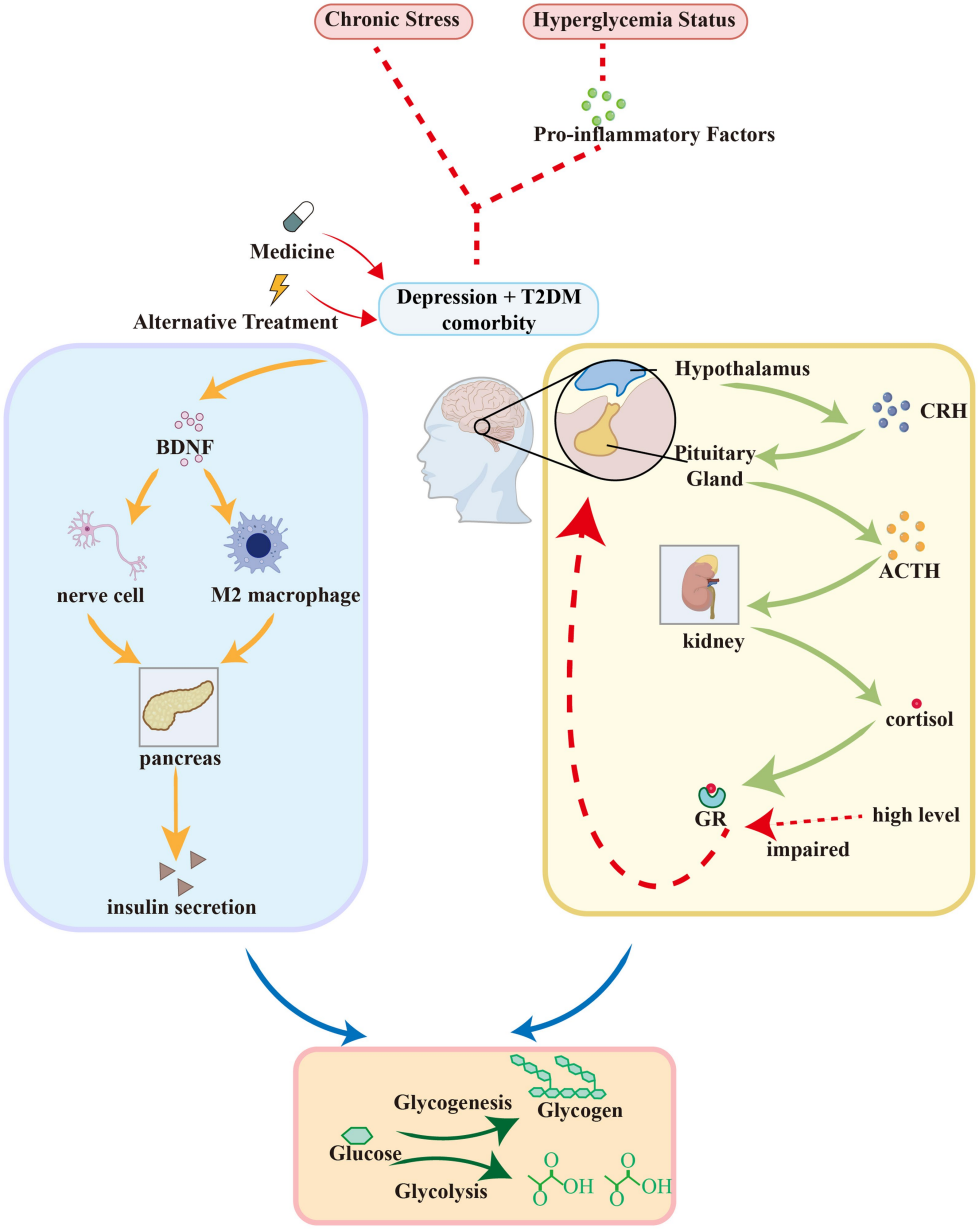
The mechanism of comorbidity through HPA axis and BDNF.

Figure 8 describes the mechanism of comorbidity through HPA axis and BDNF.

### 4.7 Integrated treatment strategy for comorbidity between depression and T2DM

#### 4.7.1 Treatment T2DM through depression

There is a bidirectional association between depression and T2DM, that depression increases the risk of developing T2DM and T2DM patients are also more likely to experience depressive symptoms (71, 73).

Antidepressants refer to a kind of psychotropic drugs primarily used to treat mental illnesses, characterized by depression (74). Antidepressant medication treatment can significantly reduce the risk of developing T2DM in patients with depression. For example, patients who use SSRIs, TCAs, heterocyclic antidepressants or other antidepressants will significantly reduce the risk of T2DM. And the risk of T2DM decreased with a certain period of time that the patients use a specific type of antidepressant (71). Selective Serotonin Reuptake Inhibitors (SSRIs) is the first artificially designed psychiatric drug commonly used to treat depression or other mental illnesses (75). SSRIs such as sertraline can not only alleviate depressive symptoms, but also indirectly improve blood glucose control by regulating neurotransmitters such as serotonin (14, 76, 77).

Psychosocial interventions such as cognitive-behavioral therapy (CBT) and supportive psychotherapy have been proven to have dual benefits for patients with T2DM and depression (25). Numerous studies have demonstrated that CBT effectively reduces depressive symptoms. Specifically, for face-to-face CBT, the mean change in symptom scores is −1.11, with a 95% credible interval ranging from −1.62 to −0.60. Hybrid CBT shows a mean change of −1.06, with a 95% credible interval of −2.05 to −0.08. In the case of multimedia CBT, the mean change is −0.59, and the 95% credible interval is −1.20 to 0.02 (78). Meanwhile, through psychosocial intervention, the patient’s fasting blood glucose, postprandial blood glucose, and glycated hemoglobin (HbA1c) were significantly reduced (25).

Another non-pharmacological treatment method for comorbidity of depression and T2DM is to change the patient’s lifestyle. Aerobic exercise can reduce binge eating behavior and improve insulin sensitivity; Avoiding a high fructose diet can reduce excessive activation of the limbic system, thereby reducing depression and metabolic disorders (77). Yoga improves depression symptoms by regulating stress hormones, such as cortisol, and enhancing autonomic nervous system function, thereby achieving blood sugar control (79, 80).

In summary, depression can be treated through multiple pathways, such as medication, psychology, lifestyle, to improve metabolic indicators, reduce the risk of complications, and enhance the quality of life of T2DM patients.

#### 4.7.2 Treatment depression through T2DM

The treatment of T2DM to improve depression is mainly carried out through the drug metformin. Metformin, as a first-line drug for the treatment of T2DM, may have a positive impact on depression through a variety of mechanisms, but there are also potential risks and dispute (81). Animal experiments have shown that metformin can reduce depressive like behavior, such as reducing immobility time in forced swimming tests and tail suspension tests in mice (82). Metformin can improve abnormal glutamatergic neurotransmission, such as reducing presynaptic glutamate release, and alleviate depressive symptoms by regulating serotonin activity and dopamine levels (72). Its anti-inflammatory properties alleviate the promoting effect of neuroinflammation on depression by inhibiting the NF-κB and Nrf2 pathways, reducing oxidative stress and inflammatory factors IL-6 and TNF-*α* (83). Also, by activating AMPK signaling pathway, metformin improves insulin resistance (IR), enhances the expression of brain-derived neurotrophic factor (BDNF), promotes hippocampal neurogenesis, and alleviates diabetes related neurodegenerative changes, and thus improves depressive symptoms (84, 85). However, long term use may increase the risk of Alzheimer’s disease (AD), especially in patients with a longer course of T2DM or comorbid depression (86), but another analysis showed that metformin reduces the risk of dementia (87), suggesting genetic or individual differences. In summary, metformin can demonstrate good efficacy in the treatment of comorbidities of depression and T2DM, but further high-quality research is needed to confirm the clinical consistency of its antidepressant effects and associated neurodegenerative risks.

### 4.8 Clinical trials in comorbidity between depression and T2DM

In the past decade, the clinical research on the comorbidity of type 2 diabetes and depression has made important progress in the aspects of epidemiological correlation, intervention mode and mechanism exploration. Through large-scale cohort study and retrospective analysis of national outpatient data, we found that there was a clear two-way correlation between type 2 diabetes and depression. An 8-year study of nearly 8million German outpatients found that patients with newly diagnosed type 2 diabetes had an increased risk of subsequent depression. This association is more significant in young people, especially under the age of 34 (88). Meanwhile, the integrated collaborative nursing model, such as team care, can improve depression symptoms and diabetes control at the same time, which is one of the most effective intervention strategies at present. However, simple improvement of depression nursing may not be enough to improve blood glucose, so comprehensive intervention is needed for diabetes self-care and lifestyle (89).

### 4.9 Advantages and limitations

This study represents the first bibliometric analysis of the comorbidity between depression and T2DM. The data extracted for this research were sourced from the WoSCC and PubMed. Among the commonly used databases, the WoSCC is highly regarded for its robust search abilities, extensive document types, and authority in academic. It includes high-quality academic journals from across the globe, each holding significant repute and authority within their respective disciplines. Consequently, utilizing the WoSCC enables researchers to access the most current and reliable academic findings.

Research on the comorbidity between depression and T2DM is of great significance. On the one hand, the phenomenon of comorbidity between the two is quite common, and studying their association can help to gain a deeper understanding of the disease mechanism and discover common pathological and physiological pathways, such as inflammatory reactions, neuroendocrine disorders, etc. (90). On the other hand, it can provide guidance for clinical treatment, which can help doctors develop more comprehensive and accurate treatment plans, intervene in both diseases, improve patients’ treatment effectiveness and quality of life, reduce disease burden, and have important value in improving patient prognosis, thus reducing the discrimination of the society against diabetes or depression patients (46, 91).

Although we made efforts to optimize search terms, it is inevitable that some outstanding papers may have been overlooked. Additionally, technical constraints related to software applications prevent simultaneous analysis of multiple databases. Moreover, bibliometric tools face challenges in differentiating between author names with identical abbreviations. Despite these limitations, our discussion incorporates recent and highly cited literature to emphasize the prominent themes and trends in the field. In future investigations, we aim to mitigate these potential shortcomings.

## 5 Conclusion

The analysis of the annual number of published documents indicates that the field of depression and T2DM comorbidity is currently in a growth period. King’s College London is the institution that has published the most documents, and the United States has published the most relevant articles. Frans Pouwer are the most prolific authors. Regarding research hotspots and frontiers, the HPA axis and BDNF are prominent topics in current investigations of this comorbidity. The integrated treatment to solve the comorbidity of depression and T2DM marks a breakthrough in the concept of chronic disease management. This reciprocal intervention model, regulating metabolic status through psychological intervention or alleviating emotional disorders through blood glucose control, brings transformative potential to the comprehensive clinical treatment or nursing framework. Overall, research on the comorbidity of depression and T2DM has been extensive and in-depth, but there is still great potential for development. Therefore, it requires cross-border collaboration and needs high-quality research results in the future.

## Data Availability

The original contributions presented in the study are included in the article/Supplementary material, further inquiries can be directed to the corresponding authors.
