# Supplementary material for: Unveiling the relationship of the comorbidity between depression and type 2 diabetes mellitus: a macro analysis and micro interpretation
Source: Front Med (Lausanne). 2026 Apr 10;13:1785271. doi: 10.3389/fmed.2026.1785271 (PMC13106598; doi:10.3389/fmed.2026.1785271)
Supplement: Supplementary file 1 [file Table_1.docx]

**Identification of studies via databases and registers**

Records removed *before screening*:

Duplicate records removed (n = 0)

Records marked as ineligible by automation tools (n = 0)

Records removed for other reasons (n = 0)

Records identified from Web of Science Core Collection (WOSCC) AND PubMed:

WOSCC (n = 6219)

PubMed (n= 436)

**Identification**

Records screened

WOSCC (n = 6219)

PubMed (n= 436)

Records excluded**

WOSCC (n = 2233)

PubMed (n= 198)

Reports sought for retrieval

WOSCC (n = 3986)

PubMed (n= 238)

Reports not retrieved

WOSCC (n = 26)

PubMed (n= 0)

**Screening**

Reports assessed for eligibility

WOSCC (n = 3960)

PubMed (n= 238)

Reports excluded:

(n = 0)

Studies included in review

(n = WOSCC (n = 3960)

PubMed (n= 238))

**Included**

*Consider, if feasible to do so, reporting the number of records identified from each database or register searched (rather than the total number across all databases/registers).

**If automation tools were used, indicate how many records were excluded by a human and how many were excluded by automation tools.

Source: Page MJ, et al. BMJ 2021;372:n71. doi: 10.1136/bmj.n71.

This work is licensed under CC BY 4.0. To view a copy of this license, visit <https://creativecommons.org/licenses/by/4.0/>
